# Putting the Onus on the Offenders: Implementing Project Cautioning And Relationship Abuse Awareness Raising Workshops for Alleged First-Time Offenders of Domestic Violence and Abuse

**DOI:** 10.1177/10778012251409171

**Published:** 2025-12-23

**Authors:** Hayley Alderson, Sara Morgan, Jessica Roy, Rachel Armitage, Ruth McGovern, William McGovern, Lydia Shrimpton, Emma Hazan, Stephanie Scott

**Affiliations:** 1 Population Health Sciences Institute, 5994 Newcastle University, Newcastle Upon Tyne, UK; 2 Centre for Population Health Sciences, 575011 University of Southampton, Southampton, UK; 3 School for Policy Studies, 1980 University of Bristol, Bristol, UK; 4 Leeds Law School, 151662 Leeds Beckett University, Leeds, UK; 5 Department of Social Work, Education and Community Wellbeing, 373117 Northumbria University, Newcastle, UK; 6 The Hampton Trust, Ashurst, UK

**Keywords:** domestic abuse, qualitative, prevention, out-of-court resolution, perpetrator workshops

## Abstract

Domestic violence and abuse (DVA) is a global public health challenge. Project Cautioning And Relationship Abuse (CARA) is an out-of-court resolution used across police forces in England and Wales for lower risk, alleged first-time DVA offenders. This article reports on qualitative interviews and focus groups with professionals (police, commissioners, and CARA facilitators and managers, *N* = 31) about their experiences of delivering CARA following its national rollout. Reflexive thematic analysis indicated strong multiprofessional support for CARA across geographical areas. Professionals recommended changes to enhance current delivery, relating to differential assessment of risk; adaptations to training/implementation; and exploring perceptions of CARA by voluntary sector organizations.

## Background

Globally, the burden of domestic violence and abuse (DVA) falls overwhelmingly and disproportionately on women and girls (Oram et al., 2022). Prevalence of DVA is higher in the majority of world countries, but also among vulnerable, seldom heard groups or those already experiencing health inequalities (Xiong et al., 2024). DVA therefore is a significant public health challenge worldwide, with a myriad of associated physical, mental health and social care consequences (Myall et al., 2024). These consequences include, but are not limited to, chronic pain, physical disability, poorer reproductive health, acquisition of HIV or other sexually transmitted infections, substance misuse and can also lead to death (World Health Organization, 2013). DVA is also linked to the exacerbation of mental health issues including posttraumatic stress disorder symptoms, anxiety, and depression (Baukaite et al., 2025).

In England and Wales, police forces will receive a call for help regarding DVA every 30 seconds (Refuge, 2024). Despite this figure, it is suggested that less than a quarter of DVA offenses are reported to the police. Even when cases of DVA are reported, research shows that it is common for victim-survivors to withdraw support for police action (Elkin, 2018; HMIC, 2014). This withdrawal of support is due to a myriad of interconnected factors including: fear of revictimization and/or retraumatization; reprisals from the perpetrator and/or their family; secondary victimization; being disbelieved and stigmatized; lack of confidence in the criminal and procedural justice system; and normalization of the DVA individuals have experienced (Evans & Feder, 2016; Hester et al., 2025). When further police action is taken, rates of conviction are low. For the year ending March 2022, of the 1.5 million reported incidents of DVA from March 2021 to 2022, 68% (910,980) were classed as a crime. However, only 7% (67,063) of those reported were referred to Crown Prosecution Service of which 72.7% (53,207) were prosecuted and of those 76.4% (40,650) were convicted. Therefore, the final conviction rate is less than 5% of the total number of DVA offenses reported to the police (Office for National Statistics, 2024). This results in victim-survivors reportedly feeling there is a lack of justice in formal systems dealing with DVA (Hester et al., 2025). The Home Office estimated that the total yearly cost of DVA to victim-survivors was £66 billion (Oliver et al., 2019). In response, the Government's 2023 strategic policing requirements paper states that “the relentless pursuit and disruption of adult perpetrators should be a national priority for police” (Home Office, 2023, p. 14).

When responding to DVA, frontline police use the Domestic Abuse, Stalking and Honour-based Violence Risk Identification Checklist (DASH) with victim-survivors. The DASH is a 27-question interview to identify “levels of risk” a victim-survivor is experiencing, with a threshold of 14 (out of 27) identifying “visible high risk” and triggering a referral to Multi-Agency Risk Assessment Conference for further consideration (SafeLives). Previous research has identified inconsistencies regarding how frontline officers complete the DASH (Myhill et al., 2023). The Domestic Abuse Risk Assessment (DARA) has been introduced in some forces to address some of the shortcomings of the DASH, however Barlow et al. (2021) argue that officers may still conceptualize risk inconsistently.

A raft of interventions exists for high-risk, high-harm perpetrators, but often these interventions are only offered once a significant amount of harm has been caused to victim-survivors. Due to limited opportunities to challenge perpetrators earlier, interventions are imposed only once behaviors are ingrained. As a direct response, measures that aim to intervene early have gathered momentum over the last few decades, including the adoption of a public health approach—similar to the approach to the management of physical violence within cities (Chandan et al., 2020). One intervention introduced as a diversion from the CJS pathway is Project Cautioning And Relationship Abuse (“CARA”). CARA was created as the first of its kind to sit as an early-intervention awareness-raising approach to policing domestic abuse. This innovative approach was developed and first implemented in 2011 and provided an opportunity for police to utilize a tiered approach and offer a conditional caution to alleged first-time adult DVA offenders classified as “standard risk” according to the DASH checklist and “professional judgment.” This conditional caution with the CARA pathway (see below) was only offered with victim-survivors’ input. Thus, CARA provides police with an alternative option to closing a case as “no further action” (NFA) or by issuing a “simple caution” (a formal warning) if Crown Prosecution Service evidence requirements are not met (College of policing, 2025). The organization responsible for delivering CARA, refers to individuals accessing CARA workshops as offenders; this is because the only assumption they can make is that there was an incident in which an individual offended, therefore, the term offender is used throughout the remainder of this article.

At its core, CARA is defined as an awareness-raising intervention for offenders, which utilizes a trauma-informed approach and motivational interviewing techniques. Facilitators and coordinators who are also trained in the CARA approach have contact with offenders. If, after meeting the eligibility criteria, offenders accept the CARA intervention, they are mandated by police to attend two 5-hr workshops held 4 weeks apart, which are delivered to a closed cohort with the same facilitators and participants in both sessions. Separate workshops are run for male and female offenders. The group sizes vary depending on the geographical area, demand and scheduling although they will consist of a minimum of three and a maximum of 12 participants. Through engagement in the workshops and feedback from victim-survivors, CARA is designed to enable offenders to understand what DVA is, the impact and consequences of DVA for the safety of a perpetrator’s (ex)partner(s) and children as well as learning tools and strategies to manage emotions and behaviors (Hampton Trust, 2024). By doing so, Project CARA recognizes the potential lifelong impacts of DVA on health, social and economic outcomes throughout the life-course (Morgan et al., 2024). Across CARA workshops, offenders are signposted onto services that support improvements in the wider determinants of their offending behavior, such as to primary care, drug and alcohol services or onto a community perpetrator program. If they fail to comply with the conditions of the caution, they may be prosecuted for the original offense. Crucially, CARA is a victim-initiated response to DVA within which National Police Chiefs’ Council Pre condition guidance states that victim-survivor views on the decision are sought and considered as to whether the offender attends the CARA workshops as an outcome. Additionally, CARA has integrated an opportunity for voluntary victim-survivor contact to take places midway between sessions.

There have been previous small-scale evaluations of Project CARA (Flowe et al., 2022; Karavias et al., 2023; Strang et al., 2017). The aim of our wider evaluation was to significantly add to this evidence base and evaluate Project CARA following its wider rollout in multiple sites across England. Further detail on the background and delivery of Project CARA can be found in Morgan et al. (2024).

The aim of this article is to present findings from the qualitative component of the evaluation to understand the perceptions and experiences of professionals in the delivery of Project CARA.

## Method

This article reports on data from a nested qualitative study conducted as part of a concurrent pragmatic mixed methods national evaluation of CARA (Morgan et al., 2024). As part of the qualitative evaluation of CARA, we conducted interviews and focus groups with professionals, victim-survivors, and perpetrators. Here, we focus on the perspectives of professionals involved in the delivery of Project CARA. Interviews were conducted between August 2022 and November 2023 and the breakdown of participants is shown in Table 1.

**Table 1.** Participant Characteristics.

Participant role	Number	Method	Gender
CARA managers	3	Interviews	2 female; 1 male
CARA co-ordinators	4	Focus group	3 female; 1 male
CARA facilitators	11	Focus groups (*n* = 3)	9 female; 2 male
Police staff	11	Interviews	5 female; 6 male
Commissioners	2	Interviews	2 female

*Note.* CARA = Cautioning And Relationship Abuse.

Research ethical approval was sought from the University of Southampton (Higher Education Institute) Ethical Committee and approved on the 1st of June 2022 (Ref: ERGO ID: 71818.A1). Recruitment materials were circulated by a gatekeeper to all CARA facilitators, CARA co-ordinators and police partners. Participants interested in taking part, notified the gatekeeper who passed the details to the research team who then contacted participants directly using the contact details provided. Interviews and focus groups were conducted by members of the research team (HA, SS, and GP). Data collection was directed by a topic guide, which reflected key themes from existing literature and developed iteratively over the course of data collection. This topic guide focused on each participant’s awareness of DVA prior to involvement with CARA, their role within CARA, perceptions of the aims and ethos of CARA, how CARA “fits” with existing interventions, how CARA responds to the needs of offenders and their (ex)partners, facilitators and barriers to implementation, outcomes for offenders, and the adaptation and sustainability of CARA. An additional question was asked of commissioners to establish what proof of effectiveness they would like to see following completion of CARA. Prior to taking part, all participants received a study information leaflet and provided written informed consent to take part in the study; confidentiality and anonymity were assured. All interviews and focus groups were recorded using video-conferencing software (MS Teams) and were done so with participants’ consent.

Transcription of audio-only data was undertaken and observational field notes were recorded. Reflexive thematic analysis involving a collaborative coding approach was used to analyze the data (Campbell et al., 2021; Clarke & Braun, 2018). This method forefronts the development of a richer, more nuanced reading of the data, rather than analysis which seeks consensus on meaning. Members of the author team contributed to the analysis of the dataset (JR, LL, WM, RA, and SM), which was led by SS and HA. The raw text of each transcript was first coded line-by-line and then systematically indexed into data tables to generate detailed descriptive themes. The author team critically challenged and discussed themes over a series of analysis meetings to identify patterns, similarities, and differences in the data and, ultimately, develop a rich and nuanced interpretation of the dataset as a whole.

## Findings

Thirty-one participants took part in an interview or focus group (see Table 1), interviews lasted 28–69 min (mean 49 min) and focus groups lasted 101–112 min (107 min). Our analysis led to four overarching themes detailed below which are contextualized by anonymized, truncated participant quotes.

### Theme 1: What Is CARA and Who Is It for?

#### Professional understanding of the CARA approach

Participants consistently articulated that Project CARA was designed to enhance the criminal justice responses to policing DVA. This was achieved by providing a tiered disposal approach and an opportunity for alleged first-time DVA offenders to engage in workshops that facilitated an increased understanding and awareness of what constituted DVA and how their abusive behavior has/will impact individuals around them.

I always say it is a bit like a driver's awareness course. For me, I'm just trying to plant that seed of look, something's happened. You've come into contact with the criminal justice system. It might be that this is a pattern … But, actually, what can we do to prevent this? (Facilitator 9, Female).

Professionals with responsibility for the delivery of CARA felt that CARA enabled an early interventionist approach to be taken. While most participants noted that CARA was an “awareness raising” not “behavior change” intervention they indicated that behavior change *was* anticipated and made possible through the provision of tools and strategies that offenders could use. For example:

It's all about that early intervention, I think, about working with domestic abuse offenders at a stage before it gets to that really serious, high-risk murder, sexual strangulation, all the higher end of things. (Coordinator 1, Female)

To assist them with strategies to change their behaviours, change their attitudes, manage themselves and their relationships, both with whomever the subject of their domestic abuse is, and that might be their current or an ex-intimate partner, or it might be other family members. That's what CARA is all about. (Manager 3, Female)

As such, CARA was recognized as multifaceted. While the provision of information and awareness raising were fundamental elements of CARA, the overarching aim appeared (to professionals) to be to elicit behavior changes to ultimately reduce rates of DVA.

It's an awareness course, with an aim to reduce offending, and to reduce the reoccurrence of offending, and thereby, by default, protect victims and reduce the number of victims. (Manager 1, Male)

Across and within participant groups, there was a recognition that CARA could act to support both offender and victim-survivors. It was felt that the provision of information to both offender and victim-survivor facilitated a co-ordinated response, promoting a consistent understanding of what constitutes a healthy relationship. Participants described their support for CARA's vision; of providing an educative intervention with potential to help offenders develop insight into their behaviors and roles. There was a perception that CARA could be significantly more impactful than the available alternatives: a simple caution or a fine, neither of which would influence behavioral insights.

It's about changing that behaviour. This isn't just about the perpetrator; this is also about the victim recognising what a healthy relationship is as well …. So, for me, it's about changing behaviour, understanding what a healthy relationship looks like for all of those involved, and hopefully deescalating a dangerous situation. (Commissioner 1, Female)

I really believe in out-of-court disposals as a way of changing people's behaviour … where they're run with proper facilitated courses that are based in psychology, I think they're better than going to court for low-level offenders, where most people will just get a fine, won't they? To actually challenge their behaviour and force them to face up to it. (Police Lead 12, Female)

#### Who Is CARA for—Identifying the right offender at the right time

The first step in the CARA journey started with police staff issuing an out-of-court resolution order (previously known as an out-of-court disposal). Participants acknowledged that this requires police staff to have a good ability to appropriately identify individuals eligible for CARA. While there was training available, there were mixed views on whether this was sufficient to upskill police staff to competently respond to out-of-court resolutions.

They get some initial training when they join up on all things out of court which will include CARA. It's not in-depth so I'd say with a high degree of confidence that most officers wouldn't have a really good understanding of what CARA is, but they would all understand that it exists and it's an option. (Police Lead 6, Male)

While Participant 6 suggested that the initial training did not provide the necessary level of information to ensure consistency, another participant felt that, while brief, the training provided all necessary and essential details to inform decision-making regarding CARA eligibility and process.

[the training was] A PowerPoint presentation, which was delivered to all frontline staff in [police locality]. Within that are slides detailing the criteria, a bit of statistical proof if you like that CARA's a positive programme, and why we should be engaging with it, and then contact details, and things like that. (Police Lead 5, Male)

A further contrasting perspective from a police lead was provided which stated that court disposals were only issued by officers who were trained and competent, therefore implying that officers would have a good understanding and the required skills to appropriately and competently allocate offenders to CARA.

We've got a significant number of sergeants who are trained, and we've got a list of them. So, we say, ‘You can only issue an out of court disposal for a domestic abuse case if you've been specially trained. You can't just go ahead and do it if you're not on our list.’ If somebody does it who's not on our list [inhales sharply] … I always check the list because it's important that they know the limitations for the … Other out of court disposals…. (Police Lead 9, Female)

#### The impact of gender when considering the appropriateness of CARA

CARA can be offered to men and women. Participants noted minimal hesitancy regarding the appropriateness of male offenders accessing the workshops. However, the likely dual identity of female offenders as victim-survivors and offenders was reflected on by CARA facilitators.

What I found is probably seven out of ten in the female group … tend to be - and this isn't me just blanking it and making a sweeping assumption - the ones I've done tend to be more than 50 per cent are actually victims of domestic abuse themselves and has retaliated in a way and that's why they are where they are. (Facilitator 1, Female)

Quite a lot of the female groups, most of them were actually victims that had been violent resisters and had often just told the police, “Oh, I've hit him.” Therefore, they’d been arrested. I remember doing about ten DASHs on one CARA, to get them all linked into victim services. (Facilitator 9, Female)

A concern was raised *that female “offenders” would be referred to CARA and this could act to potentially “revictimise genuine victims*” (Police Lead 5, Male). Participants noted that there was flexibility factored into CARA workshops to take a trauma-informed approach and provide information about risk and safety planning. Indeed, following attendance at CARA workshops, onward referrals were often made for female offenders to enable them to receive support as DVA victim-survivors.

But when you do female groups, there are actually victims in there, and you've got to be careful that whilst it's highly likely that it's been a reactionary one-off incident because abuse has been perpetrated towards them - but you've got to be careful that you don't revictimize them by having them in the room if that makes sense. (Co-ordinator 3, Male)

I think it's understanding that you've got them [victims] there in the room; let's use this as an opportunity for - what are the risky situations for you? Is it actually worth spending a bit more time on safety planning? Is it worth spending a bit more time connecting you with the right support or what have you? (Co-ordinator 2, Female)

### Theme 2: Enablers (Active Ingredients) to Successful CARA Delivery

#### A timely and proportionate response to alleged first-time offenders

As per Theme 1, CARA was felt to offer a timely response to alleged first-time offenders. Pathways which immediately divert offenders away from the justice system as CARA does, were, when implemented correctly, perceived to be more positive and proportionate than prosecutions that result in low-level penalties that do not address the offenders’ abusive behaviors.

I think the benefits are huge. If we were to send these individuals to court, most of the time they would get very little at court. It's a really good way of getting a positive intervention at an early stage, very quickly, because court, obviously, throws in other delays. (Police Lead 6, Male)

The immediacy of response and the potential to offer offenders CARA workshops within a close time proximity to the reported DVA had the potential to be impactful. Offenders had to purposefully and consciously display a level of motivation to attend CARA workshops. This was perceived as being more effective than imposing a solely administrative response, such as an “NFA” that does not require any level of responsibility, reflection, or insight.

The main benefit to the force is that instead of somebody either getting a simple caution, which … doesn't really hold much weight to it. People are now getting a two-part intervention where they also have to give their time for two Saturdays and the worse that's going to happen is, they're going to take something away. The best that's going to happen is, they're going to realise that actually they've got a lot they need to learn and might go on to get further support. (Police Lead 8, Male)

#### Facilitator attributes and the ability to create a safe environment

Facilitators unequivocally described the training they received in preparation to deliver the CARA workshops in positive terms. All CARA facilitators described attending intensive, in-person facilitator training delivered by trainers who had themselves delivered the CARA workshops. The CARA trainers could draw on their experience of working with offenders. Additionally, having an opportunity to observe the facilitators role-playing the workshop content and showcasing the desired way of delivering the content had been beneficial. For example:

Having really knowledgeable trainers that have been in the room, done it, and could answer all of my questions. (Facilitator 6, Female)

I remember it was amazing watching both of them as co-facilitators. The training was very experiential. (Co-ordinator 3, Male)

All facilitators recognized that even following the training, ongoing opportunities were available to elicit support if, as and when necessary, from CARA managers. This enables facilitators to feel supported in delivering the workshops “*They're all very open door. There's no we're not going to talk to you. If you've got a problem, talk to us*” (Facilitator 11, Female).

Once delivering the CARA workshops, CARA facilitators’ knowledge, skills, attributes, and experience were described as the critical component for successful delivery. Facilitators tended to be sessional workers with a wealth of experience obtained from working in professional environments such as: prisons and probation; sexual violence services; and victim-survivor facing organizations. Some also had lived experience of DVA, and others had previously delivered interventions for DVA offenders. A consistent belief reported was that facilitators who already had knowledge and expertise regarding DVA would be well equipped to deliver CARA.

It's helpful to have people who know about domestic abuse. They know the myths; they know the facts. You would learn as you facilitated the programmes, but it's an awful lot better if you know already. (Manager 1, Male)

During workshops, the ability of facilitators to be responsive to the dynamics of the group and manage individuals in a way that was conducive to all participants engaging was identified as important. This included facilitators being able to instill strict boundaries when required, such as not permitting attendance if participants turned up significantly late, intoxicated or displaying behaviors such as poor mental health which would not be conducive to engaging in the CARA sessions. Importantly of note, these participants were followed up and offered an alternative time to attend. Once delivering sessions, the use of motivational interviewing principles such as “expressing empathy” was described by managers and facilitators as key to CARA. The ability for professionals to portray an understanding of how offenders are feeling and facilitate opportunities to listen to their perspectives without judgment was paramount to encouraging engagement. This was closely aligned with the belief that if facilitators can successfully achieve a human connection with the offenders, it is a powerful indicator that change could happen.

The amazing power of speaking to somebody in a non-judgemental way, in an empathic non-judgemental way, I can't put it into words how much it shifts. (Coordinator 3, Male)

It's about building an understanding, and in order to do that they need to feel comfortable. They need to feel safe; they need to feel listened to and they need to feel like their contributions are valued. (Coordinator 2, Female)

CARA facilitators created a safe space to listen to participants. In this space, a therapeutic alliance with participants could be developed with facilitators using skills of empathy, congruence, and positive regard, for example:

I think, just giving an opportunity for men to be listened to, and shift that culture from push it down, don't talk about it, to actually, here you go, get some help. I think that's massive because we don't have that in our society. I think CARA addresses that really well. (Coordinator 3, Male)

Prosocial respectful relationships regardless of gender were also modeled. It was described that cofacilitators where possible model male/female dynamics, however due to the higher proportion of female facilitators, it's also common for sessions to be cofacilitated by two experienced female facilitators. This joint delivery was recognized as important with participants noting that cofacilitating with a colleague with whom they had an established working relationship had potential to result in a more dynamic and responsive workshop.

We never have two males working together, because that's what the [organisation*] have said wouldn't be appropriate, whether it's a male or a female cohort … Part of that is because, particularly with male offenders, you don't want to get into an ‘all boys together’ environment, which potentially can happen. (Manager 3, Female)

I just think when you have that connection with somebody else, you're shooting off the hip together, and that's where you're going to have better impact, more engagement, and hopefully more positive results on the outcome. (Facilitator 1, Female)

### Theme 3: Challenges of Successful Delivery of CARA

#### The challenge of managing competing priorities

While the overarching “vision” on out-of-court resolutions tended to be consistently understood and articulated, several factors impacted the ability of professional stakeholders to operationalize the delivery of CARA. These centered on how risk of harm from DVA was defined and actioned, policing partnerships, and financial constraints on delivery. At its basic level, CARA was described by police leads as an intervention that aligned with overarching visions and strategic priorities outlined in the Police and Crime Plan and contributed to their drive toward achieving positive actions in responding to DVA.

Out of court disposals in general are again forming a bigger part of the Police and Crime Plan because there's a big move to get cases away from court, and again to improve the service to victims, but also, it's about reducing reoffending. (Police Lead 1, Female)

Domestic violence is really high up on our agenda and is definitely one of our priorities and CARA is part of that toolbox. (Police Lead 6, Male)

However, there was recognition that while DVA was high up on the strategic priorities for police, police resources were often focused on offenders identified as “high risk, high harm” on the DASH (a score of 14+). Therefore, in times of limited resources, CARA could be overlooked or deprioritized.

I suppose from a domestic abuse perspective, it's still very much the culture that we're embedding in the organisation is still one very much of positive action, arrest, charge. The gold standard service for us is still charge and remand. That's the best way we can safeguard victims of domestic abuse. In terms of core messaging, that's still where we're at. (Police Lead 11, Female)

I think it's [CARA] not a priority, it's not … I suppose the cases that are coming through are low-level domestic abuse … We've got this special dispensation from the Director of Public Prosecutions to go out of court. We've got standard or medium risk or low risk, so because everything is focussed on threat, risk and harm, low comes down low. (Police Lead 9, Female)

Conflicting priorities were also described. Participants noted that while police performance is measured on arrest and conviction rates (and other statistical measurements), it was often the softer mechanisms that best supported the future reduction of offending. Such mechanisms should be focused on:

The culture that we've seen particularly in the last 12 months I think is very much one of we're back in the performance world … that's just counting beans, isn't it, whereas the quality of the outcomes is for me, and the reduction of future offending, is where we need to be focused. (Police Lead 10, Male)

#### Subjectivity in assessing levels of risk

Concerns were raised about whether different police officers in different forces would use the CARA option consistently. This had the potential, to undermine what CARA was aiming to achieve.

If you asked me today, am I confident that the same offender for the same offence in a different part of the county would get the same outcome? I'm not, that's honest. It's very subjective at the moment. Sometimes it may even be down to the knowledge of the officer or the sergeant as to what is available. (Police Lead 10, Male)

Participants were also cognizant that individual police officers may be more or less risk averse. This could be influenced by differing understandings of the nature and patterns of abuse being experienced and reported and also the ability to recognize and assess an individual's vulnerability and transient risk levels, which could impact the consistency of referrals made. However, CARA providers also recognized that as a specialist organization and the provider of the intervention, it was part of their role to ensure consistency and the relevance of CARA to the referred offenders.

Assessment is supposed to be objective; [but] it can be subjective. If I'm a little bit risk averse, I'm going to say that somebody is medium rather than say that they're low. I know there's some tension within [Area name] on who makes those assessments and how objectively they're actually made. (Manager 1, Male)

We need to be confident as a provider to be able to look at a case and go back to the officer in charge and say this is not an appropriate referral … We are the people that know this. I think that they respect the expertise that providers bring to the table in this area. (Manager 2, Female)

#### The transient nature of police forces

Relationships and building partnerships with police and CARA staff were made more challenging due to the transient nature of police staff in terms of turnover, movement, and retention. Continuous staff churn meant that awareness levels of CARA fluctuated and regularly needed to be revisited within forces. CARA managers had to retrain individual police staff and reassess levels of knowledge and collective buy-in. This was especially significant regarding senior staff working in strategic roles who had the ability to influence the level of priority afforded to the CARA intervention. This continual need to promote CARA was not only resource-intensive but had potential to further impact the consistency of approach taken by police when offering CARA as an option to DVA offenders.

There's 43 police forces in the country … You only need a chief constable to come in and change the process … and you move on. You will have a domestic abuse lead, an out-of-court disposal lead, literally about every two years … They just get into it - it takes years - and they go, ‘I'm moving on’ … That is really difficult, and that will never change. Policing, I think, is very challenging at the moment. (Manager 2, Female)

The continual rotation of police staff was a challenge for police as well because of the need for additional training and awareness raising within teams. This was problematic in terms of resources and staff time to deliver and attend training, but it also impacted the tacit knowledge and organizational memory within the team. This had potential to negatively impact the utilization and sustainability of using CARA as an available option to divert alleged first-time offenders away from the criminal justice system.

They do get trained, but we come back to the conundrum with the constant churn. You've got new people making case disposal decisions. (Police Lead 10, Male)

We're having a new cohort of student officers every three months, and there isn't a great deal of organisational memory, shall we put it. So basically, I'm having to refresh the training on each of our teams, and this is a big force, every three months. (Police Lead 1, Female)

#### Challenges of CARA workshop delivery

Another challenge identified was the inconsistency of workshop availability based on geographical location. Professionals articulated that it can be problematic to secure access to workshops for offenders if forces cover large geographic footprints or rural areas which limits offering multiple options. This had implications for offenders traveling to the workshops which, subsequently, had the potential to negatively influence levels of compliance.

The availability of workshops is always a bit of a concern because we're a large force, quite a rural force in the main … then you get people not complying because they can't get there, or they can't be bothered to get there. It's really annoying, then … it means we have to breach them, or they have to run another course, and it just becomes inefficient … Any time delay, whether it's caused by us in doing the processing, or in CARA having the courses available, we know that the bigger that time delay, the worse the compliance is. (Police Lead 6, Male)

These challenges were compounded if attendees presented with multiple complex needs which prevented them from being able to stay or fully engage in workshops. Specific examples given included offenders attending the course intoxicated, presenting as unwell due to significant mental health concerns, and language barriers.

We'll get someone turn up who speaks Romanian, and we weren't aware, and we can't tell them to go away because they don't understand any English. We just have to role play, leave, and they're leaving thinking, what am I doing, what have I done? What's going on? (Co-ordinator 3, Male)

Finally, access to financial resources to enable the sustainability and/or scale up of CARA was described. This hinged on uncertainties of what might happen when delivery was expanded and rolled out to all police forces, which most professionals expected to be the next step. Linked to future sustainability, multiple participants suggested that it would be beneficial to adapt the CARA program and deliver it as a universal educational resource to school-aged children to promote understanding regarding what constitutes an unhealthy/healthy relationship.

The biggest limitation as discussed is finances. That is where it'll either fly or fall down completely. The biggest risk to CARA in [area name] … is our ability to secure funding, to continue the pilot …. That's the point of failure for me, that if not addressed, it's just all going to fall down. (Police Lead 5, Male)

### Theme 4: Potential Impact/Benefits for Individuals With Lived Experience of DVA

#### Perceived benefit of CARA to survivors

An emphasis was placed on CARA enabling a victim-centered approach to be taken. The opportunity for offenders to engage in an out-of-court resolution had the potential to both address the offender's behavior and minimize the impact of victim-survivors having to stay engaged in lengthy CJS processes which was often verbalized as (re)traumatizing. Overwhelmingly this was perceived as providing victim-survivors with an opportunity to influence what happens following a DVA incident, therefore has potential to increase a sense of autonomy and empowerment.

The benefit to the victim? It is that they get an outcome which spares them the trauma of going through court … It also feels, I would imagine from their perspective, that finally they're having a say in what's happening to them’ So, from a victimological point of view, I think, my humble opinion is, is that at least we're starting to collaborate with victims about what's best for them. What do they want to see? What do they want to do? (Police Lead 3, Male)

The relationship between an offender and victim-survivor was recognized as multifaceted with many victim-survivors not wanting their partner arrested and/or convicted. CARA provided a meaningful chance for offenders to display an effort at reparation of relationships and to gain an understanding regarding the impact of their abuse.

I do think it gives a more victim-centric decision. It's not binary. We can resolve this; it doesn't mean a prosecution. There is an alternative, and I think that's a really good thing for the victim. Not just it gives the offender the chance, but for the victim it gives them an opportunity to rebuild a relationship knowing that their partner is willing to engage and address their behaviour. (Police Lead 10, Male)

CARA was an available option to raise awareness that DVA is not acceptable and act as a juncture for offenders to reassess their behavior. The diversionary option created a space in which offenders could reflect on their own behaviors while being supported to engage in a sympathetic but critical appraisal of their actions. It simultaneously presented an opportunity for victim-survivors to observe positive action from police without experiencing the potentially detrimental impact of criminal justice involvement for families.

So, these cases are the low risk. Maybe it's the first time, should be really the first time, and do you want a criminal record? Do you want them to go to prison? Do you want him to lose his job, not see the kids? No. … some of them want them to change behaviour, stop beating me up when you're drunk, and move on, I love you. This is what I think we bring when we do this; instead of punishment, its education, isn't it? (Police Lead 9, Female)

#### Perceived benefit of CARA for offenders

CARA facilitators recognized the multiple and complex needs that many offenders presented with and acknowledged that stigma and shame could be barriers that limited participation. Participants went further to acknowledge that, whilst not a core component of CARA, offenders would have opportunities to discuss their lived experiences within workshops. Facilitators aimed to humanize offenders within the room and recognize their intersecting needs including experiences of trauma and abuse in their own right.

The people that are actually sitting in those seats are human. They've got a story, they've got an experience, they weren't born this way, I don't buy that at all. (Facilitator 6, Female)

A lot of people who have never really dealt with what's up here [signalling their head], whether that be trauma or coming from care, people who've been failed by other adults and not had good role models are the ones we tend to see that don't really know how to be in relationships of any type sometimes. (Facilitator 3, Female)

By providing environments within which facilitators were compassionate and empathic (see theme 2, subheading “Facilitator Attributes And The Ability To Create A Safe Environment”), the aim was to encourage offenders to feel safe enough to discuss previous traumatic experiences. However, regardless of any additional complexities that were discussed, there was unanimous agreement that offenders are rightly “*never allowed to make that an excuse for the behaviour that's brought them to CARA*” (Facilitator 8, Female) and previous traumatic experiences could not be used as a justification for perpetrating abuse.

They can have other trauma. We get all kinds of disclosures in all of the different groups all the time, so it's how do we manage being empathetic and encouraging of those disclosures whilst still encouraging that accountability around the behaviour. (Co-ordinator 2, Female)

But in terms of alcohol and drugs and things like that, we tend to just make it really obvious and really straight to the point that that's not a cause for domestic abuse. That yes it was a factor, but that's not why you did it, let's look at why. (Facilitator 4, Female)

Similarly, offenders with current and ongoing needs, were not permitted by facilitators to use that to diminish responsibility for engaging in abusive behavior. Nevertheless, participants felt that disclosures regarding previous adverse experiences and contributory risk factors such as alcohol, mental health and bereavement highlighted the necessity for accessing support. The workshops could provide signposting to available support which was seen as beneficial.

If they get that help with their mental health and they get that help with the alcohol, the likelihood that the domestic abuse is going to happen again, lessens. So, I think even though it's not necessarily something specifically to do with domestic abuse, it all links. (Co-ordinator 1, Female)

When describing the perceived impact of CARA workshops, all facilitators described how they observed a tangible attitudinal shift in how offenders presented between workshops 1 and 2 resulting in offenders engaging in a fuller and more open way during their second workshop. This was frequently described as the “CARA effect” and this “effect” tended to be linked to being able to see offenders begin to recognize the impact of their behavior on others and then articulate first steps in making changes.

When you're in the room and you see it, it's so obvious. It is that CARA effect of, you see it in their body language and the way they speak to you and the way their attitude is towards what you're talking about and the way their attitude changes towards other people in the room and the entire process, I think. That is the CARA effect. (Coordinator 4, Female)

I think it's amazing. I'm yet to find a cohort where there isn't some kind of magic that happens in the room, where you just see people's perceptions shift. (Facilitator 6, Female)

#### Impact for professionals involved in CARA

Multiple CARA facilitators had a background in victim-survivor services. Of significance, they had decided to undertake training to deliver offender work in direct recognition of the potential for positive action to take place by interrupting abusive behaviors. One participant stated:

Towards the end of the time I was working as a frontline support worker at [survivor organisation 1], it suddenly struck me that almost supporting people to leave was not really addressing the issue, because we'd support people to leave and then a month later, we'd get a phone call from somebody else who was like, I met this bloke four weeks ago … It was almost like being on a hamster wheel. (Facilitator 8, Female)

Prior to delivering CARA workshops, when reflecting through a retrospective lens, facilitators felt they had placed the onus and responsibility on victim-survivors to adapt their behavior to safeguard themselves and minimize the potential for further abuse to occur. This had since shifted with participants articulating that the focus needed to be placed upon offenders to take responsibility for their actions and minimize the impact of their abuse.

I used to work for [survivor organisation 2]. So, we worked with victims, and it was all about, ‘Right, change your password on your phone, change your number, go to a refuge, go to the police, change your address, change everything. You make this change.' … I like the fact that CARA is putting the onus on the offenders, ‘You need to change this, you need to do this, you need to look at this. We'll give you these tools, we'll give you this support. So, it's more about the offender then changing rather than making the victim change their whole life to - just because they've actually been a victim of something. (Co-ordinator 1, Female)

The perspective of CARA being an approach that aimed to improve the safety of victim-survivors via working with offenders was also clearly articulated as important from a commissioning perspective also, it was stated:

That outcome of those victims. It was central to what we wanted to be able to achieve. First and foremost, we want to make people safe. (Commissioner 1, Female)

For professionals directly involved in CARA, there was a recognition that its introduction had helped to improve the morale of police staff as it provided an option for officers to offer an intervention to offenders that could be viewed as taking positive action on behalf of victim-survivors.

For the officers … they were getting a lot of job satisfaction out of using CARA because… they had victims that they were seeing time and time and time again, who just didn't want to know, and they couldn't do anything with them or for them, and then all of a sudden CARA came along and they could do something for them, so that really improved their morale. (Police Lead 1, Female)

You've got all the benefits that spin out of that for us around reduced reoffending, reduced vulnerability, and reduced harm to victims. (Police Lead 2, Male)

The success of CARA was recognized and defined in a variety of different ways depending on the organizational environment within which participants were situated. Stakeholders across professional groups articulated an unwavering belief that CARA was having a positive effect. As above, facilitators described visually observing attitudinal shifts within workshops that were perceived as positive, while police staff described receiving feedback from professional colleagues who have observed an interruption to offending behavior following completion of CARA.

I work in criminal justice services. I work quite closely with the Public Protection Unit. They say to me, ‘We'll put people on CARA, and we just never see them again’. Usually, with domestic abuse, it's one of the offences where you've got most of a revolving door. You just see the same people, every few weeks, sometimes. They absolutely loved CARA. (Police Lead 12, Female)

#### External perceptions of CARA—An opportunity for education?

Discussions regarding how participants felt CARA was perceived external to the teams directly involved in implementing it and delivering the workshops were variable. Participants across all groups thought the public perception that existed was that if offenders do not progress through the criminal justice system—they have not been adequately held accountable for their actions.

I think the balance is about right. You do get some critics that think that anybody that doesn't go to court has got away with it, and I think that's just an education that people need because I don't see it that way, at all. Actually, it's something like 75 per cent of people that go to court, walk away with a fine. (Police Lead 6, Male)

Due to CARA workshops focusing on offenders, facilitators reported encountering perceptions that CARA was a “soft approach” to responding to offenders who should receive “hard” sentences instead. Participants felt this indicated a lack of understanding of out-of-court resolutions (which might otherwise receive a caution or NFA) and of the importance of resolutions which focus upon changing the behaviors of offenders, rather than victims/survivors.

A lot of judgement. Not just in groups of that, like friends and family that have found out the work I do - why would you want to do that? For me it's so obvious, but I've been that person on the side, the professional as well on the side that's gone, why would you want to do that work. (Facilitator 6, Female)

I spoke to somebody who facilitates another domestic abuse programme, and I was quite taken aback by his thoughts on CARA, that … weren't positive. I think we're seen as, because we do the motivational interviewing, and because we don't actively challenge, and because we don't know beforehand what they did and things like that, I think we're seen as being a little bit namby-pamby almost, a bit we pamper to them, and we're too soft on them, and it doesn't work, all those sort of things. (Facilitator 4, Female)

The reaction to the provision of offender-focused work was described as “controversial,” specifically because it was felt there was a public perception that funding allocated to offender work, would be detracting from victim-survivor-focused services. Participants felt this was short-sighted and that domestic abuse work required a focus on addressing offender behavior to lessen the impact on survivors. Without taking an interventionist approach, such as CARA, the risk and harm to survivors were not being resolved.

I remember when I started doing CARA, and I was working with, I think they were both managers at the time. I did say to them, ‘I'm just trained as doing the CARA.’ They're like, ‘What's that?’ I explained to them, and they were like, why are you doing that for? I was like, ‘Well, these are victims, we can support them as much as we can, and that's great. We can do everything we can for them.’ I said, but the root of the problem is the men. If we don't support the men and understand what's going on, then we're just going to get loads of victim. We're just going to carry on having all these victims coming in, and that's what we're trying to stop. (Facilitator 11, Female)

Additionally, a discrepancy appeared to be present regarding whether external perceptions were changing. From one perspective there was a view that while initially hopefully that levels of understanding would change, a level of pessimism was present that misunderstanding regarding the motivation and purpose behind CARA would continue.

I think it will persist, to be quite honest. I thought it would go away. Once we had some decent evaluation, I thought they might start changing their minds about it and think, well, actually, this is protecting victims. (Police Lead 12, Female)

Alternatively, from a facilitator perspective, they articulated that they felt some level of change had occurred and the levels of resistance previously experienced were diminishing.

I do think we're moving forward. It's not as bad as it was, I no longer walk out of meetings and feel the knives in my back because I suggested working with perps. (Facilitator 8, Female)

## Discussion

The ability to offer an early intervention approach to alleged first-time offenders presented an opportunity to provide an educative intervention aimed at increasing awareness of DVA and how their actions impacted individuals around them. The evidence is clear that DVA takes up a considerable amount of police time (National Police Chief Council, 2023) and that how police respond to DVA has historically been considered as inadequate and/or inconsistent (Davies & Barlow, 2024). However, it is also recognized that responding to incidents of DVA is extremely challenging for police staff who are the first point of contact within the CJS (McPhee et al., 2022). As identified earlier, CARA was introduced to enhance the CJS response to policing DVA and currently there are no other comparable interventions. All participants agreed that introducing CARA into the suite of resources available to police forces provides a timely and proportionate intervention that goes beyond the provision of a fine or dismisses the DVA offence by imposing an NFA outcome.

However, our findings suggest that while CARA is available as a diversionary option, opportunities to divert offenders may be implemented inconsistently for a multitude of reasons. Some police staff recognized that eligible offenders may have been missed due to the frequent turnover of officers, with new officers not necessarily being trained in CARA. The turnover of staff impacted organizational memory and tacit knowledge (Fright et al., 2023) and contributed to inconsistent implementation across geographic police forces undermines the consistency within which CARA is utilized. Furthermore, despite eligibility criteria being developed within which assessment should be objective, our findings indicate that subjective responses and variability in how “risk averse” individual officers are when completing the DASH risk assessment can lead to further inconsistencies. Despite the DARA being introduced for frontline police officers, classification of risk may still be inconsistent (Barlow et al., 2021; Myhill et al., 2023). Moreover, while CARA closely aligns with policing priorities to respond to DVA incidents, at times the approach was described as contradictory to the traditional “arrest and charge” ethos and in instances of resource and time constraints, police focus reverted back to focusing on high-risk—high-harm offenders of DVA, with the potential to unintentionally deprioritize low-level or standard risk offenses.

It is paramount for collaborative working to take place, within which CARA managers are able to upskill officers regarding the purpose of CARA, reinforce referral process and hold police forces to account if referrals were not being administered as expected. Having appropriately trained staff to assess DVA risk and suitability to attend the CARA intervention is paramount (Myhill & Bartlett, 2024). Additionally, the training delivered to CARA facilitators and the availability of the CARA trainers to engage in continuous dialog and access support empowered facilitators to competently deliver workshops regarding sensitive topic areas in a dynamic way that was responsive to individuals present.

In relation to offender group workshops, our findings echo existing studies in the field. The acquisition of skills and information, the display of role modeling and promotion of group cohesion and support were all identified as important therapeutic factors to encourage desirable behaviors (Hamel et al., 2022). Likewise, when reporting on key themes identified by male offenders accessing DVA programs, Morrison et al. (2019) found offenders appreciated facilitators who were nonjudgmental, experienced in responding to intimate partner violence and abuse, utilized nonconfrontational strategies while also displaying honesty and challenging their behaviors. These skills and facilitator attributes were identified within our study, within which a pivotal element of success was described as having facilitators who were approachable and possessed the ability to humanize the offenders, encouraging engagement and “buy in” from attendees. CARA had the potential to act as a catalyst for offenders to recognize additional needs they may have and presented an opportunity to be signposted to other specialist services in between and/or following workshops.

The necessity for professionals, both CARA facilitators and policing partners to facilitate a shift in the necessity to focus attention on offending behavior in order to impact positively on victim-survivors was described at length. Offenders participating in DVA offender groups have been identified as externalizing their behaviors, often engaging in narratives that incorporate the words such as “only” or “just” to minimize their behavior (Kelly & Westmorland, 2016). The requirement to place responsibility back on the offender and hold them accountable for their actions was pivotal to the CARA approach. However, understanding and appreciation for CARA more widely was still perceived as being met with resistance. Specifically, it was felt that wider society and voluntary organizations perceived out-of-court resolutions as an unduly light outcome relative to the DVA offense that has taken place (Allen, 2017) and that available resources should be invested in victim-survivor facing support.

The Casey report (2023) reported that women's confidence and trust in police to respond to DVA is low and evidence shows that victim-survivors often do not wish to engage with formal criminal justice processes (McPhee et al., 2022), therefore, the ability for CARA to take a victim-centered approach and work simultaneously with victim-survivors and offenders was beneficial. The use of a conditional caution aimed to both influence offender behavior through education and prevent further harm to current and future victims (Christie et al., 2022). Taking a dual approach provided opportunity to re-educate the offender and victim-survivor on aspects of behavior that were unacceptable, safeguarding considerations and the provision of strategies to deescalate situations contributed to all parties feeling equipped to manage situations in a more acceptable way.

## Implications for Domestic Abuse Policy and Practice

A number of the key findings described above, have an implication for interventions delivered in the future regarding domestic abuse.

- For CARA to be successfully implemented organizational “buy in” from strategic leads through to front-line officers is required—this could be achieved through ongoing training and review. Each site should have a named individual to oversee and ensure CARA is effectively mobilized and delivered consistently—this could include audits of referrals made to CARA to ensure that all officers are adhering to the same referral criteria.
- Risk categorization: Steps must be taken to ensure consistency and quality assurance of categorization of risk. Those assessing risk should be aware of the impact that categorization has on victim and offender outcomes. Where there is a recategorization of risk, forces must ensure that this information is communicated to those assigning offenders to the scheme. Assigning offenders to the scheme who do not meet the risk criteria undermines the scheme's purpose and risks impacting the evaluation of outcomes.
- Currently, although the demand for CARA may be more for particular demographics, for example, for males, and those living in urban areas, CARA needs to be more inclusive of all, including females and individuals in rural areas. Future work needs to be undertaken to establish if/how CARA can be delivered successfully to individuals regardless of gender or geographic location.
- Policy partners inclusive of commissioners need to recognize the importance of Early Intervention that can address some of the identified patterns of domestic abuse.
- Aligned to the implication above, there is the potential to adapt some of the CARA intervention material for children/teenagers in school, this would be a valuable addition to the suite of resources available.
- Findings identified the importance of male/female cofacilitators when possible. This resulted in the demonstration of positive interactions between facilitators of different genders and allowed role modeling to be observed. Consideration of how this dynamic can impact future work with DVA offenders should be taken into account.
- Anecdotal reports of CARA intervention outcomes were provided. It would be beneficial to track reoffending data for CARA participants in the future.
- The “CARA effect” was repeatedly mentioned by CARA facilitators and co-ordinators. Further research to understand what factors contributed to the “CARA effect” and how could this effect be expanded in future programs should be considered.

## Conclusion

This article is the first to report on qualitative data (professional's experiences and views) of CARA, a novel out-of-court resolution for alleged first-time offenders of DVA. The article has clear limitations: it does not include the voices and experiences of victim-survivors, although this information will be included in a follow-up paper. Likewise, the article does not report on quantitative police data (about arrests, for example) from the CARA study about offender behavior. Nonetheless, the article provides clear and important findings relating to professional experiences and views of the implementation, challenges, and success of CARA. This information is critical given that the buy-in from professionals is necessary for the successful implementation of CARA in the short- and long-term.
